# HPV and cervical cancer related knowledge, awareness and testing behaviors in a community sample of female sex workers in China

**DOI:** 10.1186/1471-2458-13-696

**Published:** 2013-07-30

**Authors:** Yan Hong, Chen Zhang, Xiaoming Li, Danhua Lin, Yingjie Liu

**Affiliations:** 1 Department of Social and Behavioral Health, School of Rural Public Health, Texas A&M Health Science Center, College Station, TX, USA; 2 Rollins School of Public Health, Emory University, Atlanta, GA, USA; 3 Carman and Ann Adams Department of Pediatrics, Prevention Research Center, Wayne State University School of Medicine, 4707 St. Antoine, Suite W534, Detroit, MI, USA; 4 School of Psychology, Beijing Normal University, Beijing, China; 5 Chaoyang District Center for Disease Control and Prevention, Beijing, China

**Keywords:** Cervical cancer, HPV, Female sex workers, Prevention

## Abstract

**Background:**

Limited data suggested that the prevalence of Human Papillomavirus (HPV) among female sex workers (FSW) is much higher than in the general female population. The current study aimed to examine the HPV and cervical cancer related awareness, knowledge, and behaviors among FSW in China.

**Methods:**

A total of 360 FSW recruited from entertainment establishments in Beijing completed a self-administered survey including demographics, HPV related knowledge, and health-seeking and cervical cancer preventive behaviors.

**Results:**

Approximately 70.8% of the participants ever heard of cervical cancer, and as few as 22.1% and 13.3% ever heard of HPV and HPV vaccine, respectively. The mean score on a 7-item knowledge scale was 2.2 (SD = 2.4). Less than 10% of FSW perceived any risk of cervical cancer, and only 15.3% ever had a Pap smear. About 40.8% of FSW would accept HPV vaccine if it is free, and 21.8% would accept it even with a charge. Multivariate regression suggested that women with better knowledge of cervical cancer were more likely to have a Pap smear (aOR = 1.35); women who had tested for HIV were 11 times more likely to have a Pap smear, and women who had worked longer in commercial sex (aOR = 1.01) and had regular health check-ups (aOR = 1.95) were more likely to accept HPV vaccine.

**Conclusions:**

Our study underscores the needs for effective cervical cancer prevention programs for FSW in China and other resource-limited countries. We specifically call for cervical cancer and HPV knowledge and awareness programs and regular screening as well as HPV risk-reduction programs for these vulnerable women.

## Background

Cervical cancer, the second most common female cancer, kills more than 275,000 women annually. More than 80% of cervical cancer occurs in women in developing countries. As the most populous country, China has observed an increase in the cervical cancer incidence rate in younger females (25–44 years of age) within a context of a growing cancer burden [1,2]. For instance, the incidence rate of cervical cancer was 0.21 per 100,000 among women aged 20 to 24 from 1988 to 1992, but jumped to 1.35 per 100,000 from 1998 to 2002. Nationwide organized cervical cancer screening is not available, but a recent study reported that approximately 75,000 women developed cervical cancer and 40,000 women died from the disease each year [3].

Almost all cervical cancers are caused by human papillomavirus (HPV) infection, one of the most common sexually transmitted infections [4-6]. The prevalence of HPV is higher in less developed countries (42.2%) than in more developed regions (22.6%) and higher in women who engage in sexual risk behaviors [7,8]. For instance, having sexual intercourse at a younger age, increased number of pregnancies, increased number of sexual partners, unprotected sex, long-term use of hormonal contraceptive [9], smoking, and experience of sexual violence were all recognized as significant risk factors for HPV infection [7,8,10,11]. In addition, as HIV-infected individuals are more susceptible to a broader range of HPV genotypes [12], the high prevalence of HIV [13,14] contributes to a higher risk of HPV infection among FSW [15]. Female sex workers (FSW) are at a very high risk of HPV and cervical cancer because of their exposure to multiple risks. Limited data suggested that the prevalence of HPV in FSW is much higher than in the general female population [16-19]. Some scholars suggested that FSW are reservoirs of oncologic HPV, and cervical cancer in monogamous women develops in part as a result of transmission of these viruses to them by their husbands from FSW [19]. Despite the evidence of high prevalence of HPV in FSW, limited prevention programs are available for these vulnerable women. Assessment of knowledge, awareness, and attitudes towards cervical cancer and its prevention are necessary to design an effective prevention program. Some initiatives have been explored for the purpose of designing effective programs for the prevention of cervical cancer. For example, in some developed countries, health professionals have implemented screening programs among general women and HPV vaccination programs among school-based adolescent girls and found cervical cancer as well as HPV-related diseases have been markedly prevented [3,20-24], but programs targeting FSW have been very limited [25,26].

In addition to an increasing cancer burden, the past three decades have also witnessed the resurgence of commercial sex in China because of widening income disparities and changing sexuality [27]. An estimated four to 10 million FSW work in a complex hierarchy of sex industry [28]. Most of them are young women who have limited education, have migrated from rural areas to cities, and practice high rates of risky sexual behaviors. The prevalence of sexually transmitted infections (STI) (e.g., syphilis, gonorrhea, chlamydia, herpes simplex virus [HSV]) among them ranges from 13% to 42% [28]. Most of the research on this population has focused on HIV/STI risks, but in recent years, new studies have emerged in response to the growing concern of HPV and cervical cancer in young women. A recent study revealed that HPV prevalence among FSW in China was about 38.9%; [29] however, virtually no data were available regarding FSW’s knowledge, awareness, and behaviors related to HPV and cervical cancer. The current study aims to fill the gaps in the literature by answering the following research questions: 1) What is the level of awareness of cervical cancer, HPV, HPV vaccine among FSW? 2) What is the level of knowledge of HPV and cervical cancer among FSW? and 3) What are the factors associated with cervical cancer preventive behaviors (i.e., Pap smear behavior and acceptance of the HPV vaccine)?

## Methods

### Participants

Data in the current study were collected in 2011 from Chaoyang District in Beijing, China. Chaoyang District is the most populous district in Beijing with a population of about 3.64 million, including about 840,000 migrants. The study participants were recruited from local entertainment establishments (e.g., karaoke bars, massage parlors) that were known to the local public health agency for providing commercial sex services. Our research staff approached owners or managers or other gatekeepers of these venues to obtain their permission to conduct research on their premises. Owners or managers of a total of 15 establishments were approached, and 11 of them agreed to participate. Once we received permission, we then invited eligible women working in these establishments to participate. Participation was completely voluntary. Eligibility criteria included the following: 1) 18 years of age or older; 2) acknowledged practice of commercial sex. A small number (3% to 8% with a mean of 4%) of women we approached in each of the 11 participating establishments refused to participate. A final sample of 360 women provided written informed consent and completed a self-administered confidential survey.

### Survey procedure

The survey was conducted in separate rooms or private spaces at the establishments where women were recruited. No one was allowed to stay with participants during the survey except the interviewer, who provided assistance when necessary. All the interviewers had a prior experience working with FSW. For some FSW who never heard of cervical cancer, HPV, the HPV vaccine, or the Pap smear, the interviewer explained the terms to them before the FSW proceeded to the next question. For FSW who had reading difficulty, the interviewer read questions to them and recorded their oral responses in the survey. The survey typically took about 45 minutes to complete. Each participant received a research incentive of 20 Chinese yuan (approximately US$3.00) and reimbursement for their transportation expenses (up to US $5) upon the completion of the survey. The study protocol, including the consent process, was approved by the Institutional Review Board at Wayne State University School of Medicine in the US and Beijing Normal University in China.

### Measures

*Demographic characteristics* measures include age (in years), ethnicity (Han or ethnic minority), residency (urban or rural), marital status (unmarried, married, cohabited, divorced, or widowed), education (in years of formal schooling), length of time working in the city (in months), length of time working as a FSW (in months), and monthly income (in Chinese currency yuan). For the purpose of data analysis in the current study, we categorized ethnicity into Han and non-Han, educational attainment into less than nine-year schooling verses at least nine-year schooling, marital status into currently not married (including divorced or widowed) verses currently married (including cohabited). In addition, we divided FSW into two groups based upon the distribution of their age: younger than 25 years old and 25 years of age and older.

*Health-seeking behaviors* were measured using two questions regarding whether the FSW ever had regular health check-up or HIV testing (1 = yes, 0 = no).

*Awareness.* The participants were asked whether they ever heard of cervical cancer, HPV, and the HPV vaccine (1 = ever vs. 0 = never); they were also asked to rate their *perceived likelihood of getting cervical cancer* (e.g., very likely, likely, not likely, not likely at all). For the purpose of data analysis, we assigned respondents who answered “very likely” and “likely” into “high perceived risk of cervical cancer” group; otherwise, we assigned them into “low perceived risk of cervical cancer” group.

*Knowledge* of cervical cancer prevention and HPV was measured using a 7-item scale. The scale was developed based upon existing literatures [30,31] as well as a WHO guideline on HPV and HPV vaccines [15]. The knowledge scale included questions regarding possible transmission routes of HPV (e.g., sexual transmission), risk factors of cervical cancer (e.g., early sex debut; HPV infection), and preventive measures of cervical cancer (e.g., annual Pap smear, HPV vaccination). For each question, the response was coded as 1 = correct answer or 0 = incorrect answer. The composite score of the scale was calculated by summing correct answers to all seven questions with a higher score indicating a higher level of cervical cancer knowledge. The Cronbach alpha for the seven items was 0.87 in the current sample.

Questions on *cervical cancer preventive behavior* included whether they ever had a Pap smear (1 = yes; 0 = no) and whether they would have an HPV vaccination (yes if free, yes even with a charge, and no). Respondents who answered “yes if free” or “yes even with a charge” were categorized into “1 = willing to have an HPV vaccine” group; otherwise, they are assigned into “0 = unwilling to have an HPV vaccine”.

### Data analysis

First, we conducted an exploratory data analysis to examine the distribution of key demographic and background variables of interest in the current study. The variables of interest included FSW’s demographic information and health-seeking behaviors. Second, we employed Chi-square test and ANOVA to compare women’s awareness as well as knowledge and behaviors with regard to cervical cancer and HPV. For analysis with knowledge, we only included responses from FSW who were aware of cervical cancer or HPV. Third, univariate logistic models and multivariate logistic models were employed to examine the association of cervical cancer-related preventive behaviors with key demographic and awareness variables. The measures of preventive behaviors (willingness of having an HPV vaccine and had a Pap smear) were dichotomized to serve as dependent variables in the logistic regression models. Demographic and background variables that were significantly associated with the preventive behaviors (p < .05) in the univariate analyses were included in the multivariate models as independent variables. Unadjusted odds ratios (ORs) and adjusted odds ratios (aORs) from the logistic regression models and their 95% confidence intervals (95% CIs) were used to depict the relationship between cervical cancer-related preventive behaviors and independent variables. All statistical analyses were performed using SPSS 18.0.

## Results

Table 1 presents demographic characteristics and health-seeking behaviors of the sample. The mean age of the FSW was 25.1 years (SD = 3.5, range:18–36 years old), a majority (91.1%) of them were ethnic Han, 64.2% came from rural areas, 61.9% were currently not married, and 58.1% had less than nine years of schooling. On average, they have worked in the city for 50.4 months (SD = 34.4) and worked as a FSW for 20.5 months (SD = 18.6). Their monthly income was about 4,440 yuan (SD = 2,260). Approximately 17.1% of FSW ever had HIV testing, but only 9.1% had regular health check-ups.

**Table 1:** Demographics of 360 study participants (N = 360)

**Variable**	**Value**
**Age** (mean + SD), yr	25.1 (3.47)
**Ethnicity**	
Han	91.1%
Non-Han	8.9%
**Residency**	
Urban	35.8%
Rural	64.2%
**Marriage status**	
Currently married	38.1%
Not currently married	61.9%
**Education**	
Nine years schooling or less	58.1%
More than nine years of schooling	41.9%
**Length of working in city** (Mean, SD), month	50.4 (34.4)
**Length of being FSWs** (Mean, SD), month	20.5 (18. 6)
**Monthly income** (Mean, SD), 100 yuan^a^	44.4 (22.6)
**Had regular health check up**	32 (9.1%)
**Ever had HIV testing**	59 (17.1%)

Note: ^a^ at the time of study, 6.5 yuan is equivalent to 1 US $.

As shown in Table 2, 70.8% of FSW ever heard of cervical cancer, 22.1% heard of HPV, and 13.3% heard of the HPV vaccine. Less than 10% of FSW perceived any risk of cervical cancer, and only 15.3% ever had a Pap smear. About 62% of FSW were willing to have the HPV vaccine, including 40.8% were willing if it is free and 21.4% were willing even if they have to pay. The distribution of knowledge of cervical cancer and HPV was examined in Table 3. Their responses to the 7-item knowledge scale varied, with total scores ranging from 0 to 7 and a mean of 2.3 (SD = 2.3). Older FSW were less aware of cervical cancer but for those who were aware, they had better knowledge (see Tables 2 and 3).

**Table 2:** FSW’s awareness, perceptions, and behaviors on cervical cancer and HPV (N = 360)

		**Age group**		
**Item**	**N (%)**	**18-24 years old (N = 163)**	**25 years and above (N = 197)**	**p-value**
Ever heard of cervical cancer	247(70.8%)	122 (77.7%)	125 (65.1%)	0.013
Ever heard of HPV	76 (22.1%)	32 (20.5%)	44 (23.4%)	0.602
Ever heard of HPV vaccine	46 (13.3%)	15 (9.4%)	31 (16.5%)	0.058
Perceived at risk of cervical cancer	36 (10.0%)	17 (10.4%)	19 (9.6%)	0.861
Ever had pap smear	54(15.3%)	19 (11.7%)	35 (18.2%)	0.103
Wiling to have HPV vaccine				0.180
*Willing even if not for free*	73 (21.4%)	26 (16.9%)	47 (25.1%)	
*Willing if free*	139 (40.8%)	66 (42.9%)	73 (39.0%)	
*No willing*	129 (37.8%)	62 (40.3%)	67 (35.8%)	

**Table 3:** **Knowledge on HPV infection, Pap smear, and cervical cancer (N = 256)**^**a**^

	**Overall distribution (N = 256)**			**Correct answers by age group, N (%)**		
**Knowledge item (correct response)**	**Yes**	**No**	**Don’t Know**	**18-24 years old (N = 125)**	**25 years and above (N = 131)**	**p-value**
HPV is transmitted by sexual activities (yes)	77 (30.4%)	45 (17.8%)	131 (51.8%)	33 (26.4%)	44 (33.6%)	0.223
If a woman has an earlier sexual debut or has more sexual partners, she is more likely to have cervical cancer (yes)	109 (42.9%)	39 (15.4%)	106 (41.7%)	48 (38.4%)	61 (46.6%)	0.207
HPV infection increased the likelihood of getting cervical cancer (yes)	91 (35.5%)	13 (5.1%)	150 (59.1%)	36 (28.8%)	55 (42.0%)	0.036
Women should do pap smear once a year (yes)	106 (41.9%)	13 (5.1%)	134 (53.0%)	43 (34.4%)	63 (48.1%)	0.031
Women older than 21 yrs or have more than 3 yrs of sexual activities, should do pap smear every year (yes)	105 (41.5%)	12 (4.7%)	136 (53.8%)	43 (34.4%)	62 (47.3%)	0.042
The best time for HPV vaccine is age 11 to 12 yrs old (yes)	31 (12.3%)	35 (13.9%)	186 (73.8%)	11 (8.8%)	20 (15.3%)	0.128
If a woman receives HPV vaccine, she doesn’t need to do pap smear any more (no)	32 (12.6%)	65 (25.7%)	156 (61.7%)	30 (24.0%)	35 (26.7%)	0.668
Mean score^b^ (SD, Range)	2.3 (2.3, 0–7)			2.0 (2.3,0-7)	2.6 (2.3,0-7)	0.026

Notes: ^a^ Only included women who were aware of cervical cancer and HPV (n = 256) in the knowledge assessment; ^b^ Mean score is the sum of the correct responses to the seven items evaluating HPV knowledge.

Table 4 presents the outcomes of univariate logistic regression and multivariate logistic regression with regard to the factors associated with two key preventive behaviors—ever had a Pap smear and willingness to have an HPV vaccine. We found that, while controlling for confounders, cervical cancer-related knowledge (aOR = 1.35, 95% CI = 1.15, 1.59) and ever had HIV testing (aOR = 11.14, 95% CI = 5.36, 23.15) were the two significant correlates for Pap smear; having a regular health check-up (aOR = 1.95, 95% CI = 1.01, 3.77) and working longer in commercial sex (aOR = 1.01, 95%CI = 1.00, 1.03) were significant correlates for acceptance of HPV vaccine.

**Table 4:** Multivariate predictors of having Pap smear and acceptance of HPV vaccine among FSW (N = 360)

		**Had a pap smear**				**Willing to have an HPV vaccine**^**a**^			
		**Unadjusted OR (95%CI)**^**d**^	**p-value**	**Adjusted OR**^**b **^**(95%CI) **^**d**^	**p-value**	**Unadjusted OR (95%CI)**^**d**^	**p-value**	**Adjusted OR**^**b **^**(95%CI)**^**d**^	**p-value**
**Age group**	25 years of age and older	1.64 (0.90,2.99)	0.108	--	n/a	1.20 (0.79,1.83)	0.391	--	n/a
	Younger than 25 years old	1.00		--		1.00		--	
**Ethnicity**	Han	0.35 (0.08,1.53)	0.164	--	n/a	1.37 (0.64,2.93)	0.419	--	n/a
	Non-Han	1.00		--		1.00		--	
**Residency**	Rural	0.88 (0.48,1.62)	0.678	--	n/a	0.86 (0.56,1.34)	0.508	--	n/a
	Urban	1.00		--		1.00		--	
**Marital status**	Currently married	0.95 (0.52,1.73)	0.867	--	n/a	0.88 (0.57,1.35)	0.555	--	n/a
	Not current married	1.00		--		1.00		--	
**Education**	At least nine-year schooling	0.59 (0.32,1.09)	0.094	--	n/a	0.96 (0.63,1.47)	0.841	--	n/a
	Less than nine-year schooling	1.00		--		1.00		--	
**Length of working (months)**^**c**^		1.01 (1.00,1.02)	0.004	1.01 (1.00,1.02)	0.320	1.01 (1.00,1.01)	0.044	1.00 (1.00,1.01)	0.392
**Length of working in commercial sex (months)**^**c**^		1.03 (1.02,1.04)	0.000	1.01 (0.99,1.04)	0.224	1.02 (1.01,1.03)	0.005	1.01 (1.00,1.03)	0.044
**Monthly income (per 100Yuan)**^**c**^		1.02 (1.01,1.03)	0.005	1.00 (0.99,1.02)	0.605	1.00 (0.99,1.01)	0.437	--	n/a
**Perceived risk of cervical cancer**	High-perceived	1.15 (0.45,2.91)	0.768	--	n/a	0.86 (0.43,1.72)	0.669	--	n/a
	Low perceived	1.00		--		1.00		--	
**Knowledge on HPV & cervical cancer**^**c**^		1.52 (1.32,1.76)	0.000	1.35 (1.15,1.59)	0.000	1.08 (0.98,1.18)	0.133	--	n/a
**Had regular health check-up**	Yes	0.51 (0.24,1.12)	0.094	--	n/a	2.09 (1.09,4.00)	0.027	1.95 (1.01,3.77)	0.047
	No	1.00		--		1.00		1.00	
**Had HIV testing**	Yes	13.38 (6.88,26.02)	0.000	11.14 (5.36,23.15)	0.000	1.58 (0.87,2.85)	0.130	--	n/a
	No	1.00		1.00		1.00		--	

Note: ^a^ Willingness to take an HPV vaccine combines those are willing to take the vaccine if free and those willing to take vaccine even if not for free; ^b^ Adjusted models only included statistically significant variables in the unadjusted analyses; ^c^ Serving as continuous variables in the regression models; ^d^ 95%CI means 95% confidence interval.

## Discussion

This is one of the first studies that measures cervical cancer and HPV- related knowledge, awareness, and behaviors among FSW in China. Our data revealed that FSW in China had low levels of knowledge of cervical cancer, HPV, and the HPV vaccine. Few FSW perceived themselves at risk for cervical cancer despite their high exposure to multiple sex partners and high rates of unprotected sex. Only a small portion of FSW ever had a Pap smear, but many were willing to take the HPV vaccine. The level of cervical cancer- and HPV-related knowledge among FSW was lower than that of FSW in other countries, such as Thailand and Peru [25,26]. as well as general urban Chinese women [32]. For example, a recent study among Peruvian FSW showed that 69.0% of participants were aware of the transmission route of HPV, compared to 26.3% in the current study [25]. Another study conducted in Thailand revealed that more than half of FSW knew the possible consequences of HPV infection, but less than one third of FSW in the current study were aware of them [26].

Our data provide useful information for further research and policy making. As China’s cancer burden continues to grow, more attention has been directed toward effective prevention programs [33]. Overall cervical cancer mortality has been declining, but the declining trends showed only for older women; younger women showed an increasing trend [1]. Given the large population and limited resources in most areas of China, identifying the most at-risk populations and designing targeted prevention interventions are most efficient.

To date, no national cancer prevention program is available in China despite several national and international efforts working towards this goal [33,34]. Most urban women have access to cancer screening through their employer-sponsored health insurance, but rural women have had access to free cervical cancer screening under a government-sponsored program only since 2009 [35]. The majority of FSW in China are rural-to-urban migrants and not covered by any health insurance; furthermore, the stigma against commercial sex has placed them in “double jeopardy” [28]. In a study to examine barriers to HIV testing, Hong and colleagues found that perceived stigma and denial of risk were the major reasons for not seeking testing [36]. The literature documents very high rates of HPV infection in FSW, including recent data from China showing an HPV prevalence of 39% in FSW [11,16-19,29,32,37], which stands in sharp contrast to the very low level of awareness, knowledge, and perceived risk of cervical cancer in FSW in China. As demonstrated in the current study, only 15% of FSW in China ever had a Pap smear compared to 70% of their counterparts in Thailand [26]. We therefore call for policies and programs to promote cervical cancer awareness and screening in this population in China.

Empirical studies, including the ones conducted in China, showed that early screening can efficiently reduce cervical cancer mortality [23,24]. Several model-based cost-effectiveness analyses suggested that HPV DNA screening plus HPV vaccine in certain high-risk population is the most effective approach to control cervical cancer [38]. FSW are at high risk for cervical cancer, yet they are highly mobile and severely stigmatized; thus, prevention efforts to reduce cervical cancer morbidity and mortality in this population deserve thoughtful planning and implementation. First, given the HPV vaccine is designed to be prophylactic, it is most effective for young women without previous or current infection with vaccine-related HPV genotypes [15]. However, the already-high HPV prevalence among FSW prevents the vaccine from being effective among this population. Therefore, it merits further research to explore the feasibility of vaccinating young women, especially rural women. Second, as a positive HPV test (especially HPV DNA screening) will only tell the provider that a FSW is HPV infected but not necessarily that she has cervical dysplasia [29], we thus recommend that a positive HPV test for a FSW should be used as a proxy for cervical abnormality and a cytology examination needs to be performed on the HPV-positives. Third, our data indicated that ever had HIV testing was the most significant predictor of a Pap smear (aOR = 11). China has established a comprehensive HIV voluntary counseling and testing (VCT) program, and the service has been scaled up to more than 6,000 clinics among all provinces nationwide [39,40]. We thus suggest that cervical cancer screening and counseling be integrated into the existing VCT practice for women. Finally, FSW constitute a highly heterogeneous population, and their risk behaviors vary considerably across age groups and working venues; any prevention intervention programs must be tailored to the subgroups to improve efficacy.

Our study has several limitations. First, our survey was based on a closed-ended questionnaire; open-ended questions might have gauged better cervical cancer-and HPV-related awareness and perceptions, especially when no prior data were available from this population. Second, our study was based on a cross-sectional survey, and the correlation observed in our data could not be interpreted as a causal relationship. Finally, our sample was a convenience community sample which may limit our ability to generalize the findings to FSW populations in other areas of China.

## Conclusion

Our study examines HPV and cervical cancer related knowledge, awareness and behaviors among FSW in China. Despite being at high risk for HPV infection, they have very low levels of knowledge and awareness of cervical cancer and HPV; very low rates of Pap smears and perceived risks have increased their actual risks. These findings underscore the necessity and urgency of targeted intervention. We call for culturally appropriate programs to promote awareness of HPV and cervical cancer and to provide access to low or no-cost cervical cancer screening for all FSW and low-cost HPV vaccine for young FSW.

## Competing interests

The authors declare that they have no competing interests.

## Authors’ contributions

YH made substantial contribution to conception and design as well as manuscript development. CZ performed data analysis and manuscript development. XL supervised the project, and participated in its design and coordination and helped to draft the manuscript. DL and YL collected research data. All authors read and approved the final manuscript.

## Pre-publication history

The pre-publication history for this paper can be accessed here:

http://www.biomedcentral.com/1471-2458/13/696/prepub

## References

[B1] Lei T Mao W Lei T Dai L Fang L Chen W Zhang S Incidence and mortality trend of cervical cancer in 11 cancer registries of China Chin J Cancer Res 2011 23 1 10 14 10.1007/s11670-011-0010-x 23467450 PMC3587525

[B2] Yang L Parkin DM Ferlay J Li L Chen Y Estimates of cancer incidence in China for 2000 and projections for 2005 Cancer Epidemiol Biomarkers Prev 2005 14 1 243 250 15668501

[B3] Arbyn M Walker A Meijer CJ HPV-based cervical-cancer screening in China Lancet Oncol 2010 11 12 1112 1113 10.1016/S1470-2045(10)70262-X 21075053

[B4] Bosch FX Lorincz A Munoz N Meijer CJ Shah KV The causal relation between human papillomavirus and cervical cancer J Clin Pathol 2002 55 4 244 265 10.1136/jcp.55.4.244 11919208 PMC1769629

[B5] Munoz N Bosch FX de Sanjose S Tafur L Izarzugaza I Gili M Viladiu P Navarro C Martos C Ascunce N The causal link between human papillomavirus and invasive cervical cancer: a population-based case–control study in Colombia and Spain Int J Cancer J International du cancer 1992 52 5 743 749 10.1002/ijc.2910520513 1330933

[B6] Walboomers JM Jacobs MV Manos MM Bosch FX Kummer JA Shah KV Snijders PJ Peto J Meijer CJ Munoz N Human papillomavirus is a necessary cause of invasive cervical cancer worldwide J Pathol 1999 189 1 12 19 10.1002/(SICI)1096-9896(199909)189:1<12::AID-PATH431>3.0.CO;2-F 10451482

[B7] Munoz N Castellsague X Bosch FX Tafur L de Sanjose S Aristizabal N Ghaffari AM Shah KV Difficulty in elucidating the male role in cervical cancer in Colombia, a high-risk area for the disease J Natl Cancer Inst 1996 88 15 1068 1075 10.1093/jnci/88.15.1068 8683638

[B8] Winer RL Hughes JP Feng Q Xi LF Lee SK O'Reilly SF Kiviat NB Koutsky LA Prevalence and risk factors for oncogenic human papillomavirus infections in high-risk mid-adult women Sex Transm Dis 2012 39 11 848 856 10.1097/OLQ.0b013e3182641f1c 23064533 PMC3476060

[B9] Castellsague X Munoz N Chapter 3: Cofactors in human papillomavirus carcinogenesis–role of parity, oral contraceptives, and tobacco smoking J Natl Cancer Inst Monogr 2003 31 20 28 12807941

[B10] Chandeying V Garland SM Tabrizi SN Prevalence and typing of human papilloma virus (HPV) among female sex workers and outpatient women in southern Thailand Sex Health 2006 3 1 11 14 10.1071/SH05019 16607969

[B11] Juarez-Figueroa LA Wheeler CM Uribe-Salas FJ Conde-Glez CJ Zampilpa-Mejia LG Garcia-Cisneros S Hernandez-Avila M Human papillomavirus: a highly prevalent sexually transmitted disease agent among female sex workers from Mexico City Sex Transm Dis 2001 28 3 125 130 10.1097/00007435-200103000-00001 11289192

[B12] Strickler HD Burk RD Fazzari M Anastos K Minkoff H Massad LS Hall C Bacon M Levine AM Watts DH Natural history and possible reactivation of human papillomavirus in human immunodeficiency virus-positive women J Natl Cancer Inst 2005 97 8 577 586 10.1093/jnci/dji073 15840880

[B13] Baral S Beyrer C Muessig K Poteat T Wirtz AL Decker MR Sherman SG Kerrigan D Burden of HIV among female sex workers in low-income and middle-income countries: a systematic review and meta-analysis Lancet Infect Dis 2012 12 7 538 549 10.1016/S1473-3099(12)70066-X 22424777

[B14] Platt L Grenfell P Fletcher A Sorhaindo A Jolley E Rhodes T Bonell C Systematic review examining differences in HIV, sexually transmitted infections and health-related harms between migrant and non-migrant female sex workers Sex Transm Infect 2012 89 4 311 319 10.1136/sextrans-2012-050491 23112339 10.1136/sextrans-2012-050491

[B15] WHO Technical information for policy-makers and health professionals 2007 Genava: Department of Immunization VaB

[B16] Mak R Van Renterghem L Cuvelier C Cervical smears and human papillomavirus typing in sex workers Sex Transm Infect 2004 80 2 118 120 10.1136/sti.2002.003749 15054172 PMC1744807

[B17] Montano SM Hsieh EJ Calderon M Ton TG Quijano E Solari V Zunt JR Human papillomavirus infection in female sex workers in Lima, Peru Sex Transm Infect 2011 87 1 81 82 10.1136/sti.2010.043315 20813720 PMC3501137

[B18] Smith JS Van Damme K Randrianjafisamindrakotroka N Ting J Rabozakandraina T Randrianasolo BS Raharinivo M Zanasaotra S Hobbs M Rinas A Human papillomavirus and cervical neoplasia among female sex workers in Madagascar Int J Gynecol Cancer 2010 20 9 1593 21370602

[B19] Thomas DB Ray RM Kuypers J Kiviat N Koetsawang A Ashley RL Qin Q Koetsawang S Human papillomaviruses and cervical cancer in Bangkok. III. The role of husbands and commercial sex workers Am J Epidemiol 2001 153 8 740 748 10.1093/aje/153.8.740 11296145

[B20] Fylan F Screening for cervical cancer: a review of women’s attitudes, knowledge, and behaviour Br J Gen Pract 1998 48 433 1509 10024713 PMC1313202

[B21] Giles M Garland S A study of women’s knowledge regarding human papillomavirus infection, cervical cancer and human papillomavirus vaccines Aust N Z J Obstet Gynaecol 2006 46 4 311 315 10.1111/j.1479-828X.2006.00598.x 16866792

[B22] Pruitt SL Parker PA Peterson SK Le T Follen M Basen-Engquist K Knowledge of cervical dysplasia and human papillomavirus among women seen in a colposcopy clinic Gynecol Oncol 2005 99 3 S236 S244 10.1016/j.ygyno.2005.07.095 16150483

[B23] Jun JK Choi KS Jung KW Lee HY Gapstur SM Park EC Yoo KY Effectiveness of an organized cervical cancer screening program in Korea: results from a cohort study Int J Cancer Journal international du cancer 2009 124 1 188 193 10.1002/ijc.23841 18785204

[B24] Anttila A Pukkala E Soderman B Kallio M Nieminen P Hakama M Effect of organised screening on cervical cancer incidence and mortality in Finland, 1963–1995: recent increase in cervical cancer incidence Int J Cancer Journal international du cancer 1999 83 1 59 65 10.1002/(SICI)1097-0215(19990924)83:1<59::AID-IJC12>3.0.CO;2-N 10449609

[B25] Brown B Carcamo C Blas MM Valderrama M Halsey N Peruvian FSWs: understanding HPV and barriers to vaccination Vaccine 2010 28 49 7743 7747 10.1016/j.vaccine.2010.09.063 20932498

[B26] Kietpeerakool C Phianmongkhol Y Jitvatcharanun K Siriratwatakul U Srisomboon J Knowledge, awareness, and attitudes of female sex workers toward HPV infection, cervical cancer, and cervical smears in Thailand Int J Gynaecol Obstet 2009 107 3 216 219 10.1016/j.ijgo.2009.07.023 19716556

[B27] Ruan FF Sex in China: Studies in Sexology in Chinese Culture (Perspectives in Sexuality) 1991 New York: Spinger

[B28] Hong Y Li X Behavioral studies of female sex workers in China: a literature review and recommendation for future research AIDS Behav 2008 12 4 623 636 10.1007/s10461-007-9287-7 17694431

[B29] Li HM Liang GJ Yin YP Wang QQ Zheng ZJ Zhou JJ Jiang N Tan GJ Wang B Chen XS Prevalence and genotype distribution of human papillomavirus infection among female sex workers in Guangxi, China: Implications for interventions J Med Virol 2012 84 5 798 803 10.1002/jmv.23264 22431029

[B30] Kamzol W Jaglarz K Tomaszewski KA Puskulluoglu M Krzemieniecki K Assessment of knowledge about cervical cancer and its prevention among female students aged 17–26 years Eur J Obstet Gynecol Reprod Biol 2013 166 2 196 203 10.1016/j.ejogrb.2012.10.019 23141797

[B31] Perrotte N Gomez A Mason G Stroup D An assessment of knowledge, attitudes and behaviour regarding the human papillomavirus West Indian Med J 2012 61 1 58 63 22808567

[B32] Li J Li LK Ma JF Wei LH Niyazi M Li CQ Xu AD Wang JB Liang H Belinson J Knowledge and attitudes about human papillomavirus (HPV) and HPV vaccines among women living in metropolitan and rural regions of China Vaccine 2009 27 8 1210 1215 10.1016/j.vaccine.2008.12.020 19135493

[B33] Li J Kang LN Qiao YL Review of the cervical cancer disease burden in mainland China Asian Pacific J Cancer Prevention: APJCP 2011 12 5 1149 1153 21875257

[B34] Wen C China’s plans to curb cervical cancer Lancet Oncol 2005 6 3 139 141 10.1016/S1470-2045(05)01761-4 15737830

[B35] Women’s health in rural China Lancet 2009 374 9687 358 http://www.ncbi.nlm.nih.gov/pubmed/19647592 19647592 10.1016/S0140-6736(09)61394-5

[B36] Hong Y Zhang C Li X Fang X Lin X Zhou Y Liu W HIV testing behaviors among female sex workers in Southwest China AIDS Behav 2012 16 1 44 52 10.1007/s10461-011-9960-8 21538081 PMC8208227

[B37] Vallès X Murga GB Hernández G Sabidó M Chuy A Lloveras B Alameda F de San JS Bosch FX Pedroza I High prevalence of human papillomavirus infection in the female population of Guatemala Int J Cancer 2009 125 5 1161 1167 10.1002/ijc.24444 19415744

[B38] Levin CE Sellors J Shi JF Ma L Qiao YL Ortendahl J O'Shea MK Goldie SJ Cost-effectiveness analysis of cervical cancer prevention based on a rapid human papillomavirus screening test in a high-risk region of China Int J Cancer J International du cancer 2010 127 6 1404 1411 10.1002/ijc.25150 20049838

[B39] Wu Z Sullivan SG Wang Y Rotheram-Borus MJ Detels R Evolution of China’s response to HIV/AIDS Lancet 2007 369 9562 679 690 10.1016/S0140-6736(07)60315-8 17321313 PMC7137740

[B40] Nearly 6000 Voluntary Counseling Testing Clinics in China Have Been Established[http://news.xinhuanet.com/newscenter/2008-12/01/content_10439707.htm]

